# Disruption of Notch signaling aggravates irradiation-induced bone marrow injury, which is ameliorated by a soluble Dll1 ligand through Csf2rb2 upregulation

**DOI:** 10.1038/srep26003

**Published:** 2016-05-18

**Authors:** Juan-Juan Chen, Xiao-Tong Gao, Lan Yang, Wei Fu, Liang Liang, Jun-Chang Li, Bin Hu, Zhi-Jian Sun, Si-Yong Huang, Yi-Zhe Zhang, Ying-Min Liang, Hong-Yan Qin, Hua Han

**Affiliations:** 1 Department of Hematology, Tangdu Hospital, Fourth Military Medical University, Xi’an, 710038, China; 2 Department of Medical Genetics and Developmental Biology, Fourth Military Medical University, Xi’an, 710032, China; 3 Department of Hematology, Xijing Hospital, Fourth Military Medical University, Xi’an, 710032, China

## Abstract

Physical and chemical insult-induced bone marrow (BM) damage often leads to lethality resulting from the depletion of hematopoietic stem and progenitor cells (HSPCs) and/or a deteriorated BM stroma. Notch signaling plays an important role in hematopoiesis, but whether it is involved in BM damage remains unclear. In this study, we found that conditional disruption of RBP-J, the transcription factor of canonical Notch signaling, increased irradiation sensitivity in mice. Activation of Notch signaling with the endothelial cell (EC)-targeted soluble Dll1 Notch ligand mD1R promoted BM recovery after irradiation. mD1R treatment resulted in a significant increase in myeloid progenitors and monocytes in the BM, spleen and peripheral blood after irradiation. mD1R also enhanced hematopoiesis in mice treated with cyclophosphamide, a chemotherapeutic drug that induces BM suppression. Mechanistically, mD1R increased the proliferation and reduced the apoptosis of myeloid cells in the BM after irradiation. The β chain cytokine receptor Csf2rb2 was identified as a downstream molecule of Notch signaling in hematopoietic cells. mD1R improved hematopoietic recovery through up-regulation of the hematopoietic expression of Csf2rb2. Our findings reveal the role of Notch signaling in irradiation- and drug-induced BM suppression and establish a new potential therapy of BM- and myelo-suppression induced by radiotherapy and chemotherapy.

Radiotherapy has been widely used in hematopoietic neoplasms and malignant solid tumors. This treatment, as well as accidental irradiation or the intake of toxic chemicals, damages hematopoietic stem and progenitor cells (HSPCs) and the hematopoietic microenvironment^1^,^2^. Consequently, myeloid cells, a rapidly replenishing cell population primarily involved in innate immunity, are depleted, thus resulting in elevated susceptibility to infections from pathogenic or commensal microbes. Therefore, it will be of great significance to promote the recovery of HSPCs and myeloid cells to avoid neutropenia, thrombocytopenia and anemia, which increase the risk of infection, hemorrhage and death after irradiation^3^,^4^. Various radio-mitigators such as antioxidants, antiapoptotic cytokines, and hematopoietic growth factors have been developed to treat myelo-suppression by stimulating HSPC proliferation and differentiation^4^,^5^,^6^.

The self-renewal of HSPCs requires multiple intrinsic mechanisms and extrinsic molecular signals from the bone marrow (BM) microenvironment, which has been defined as hematopoietic niches, including endosteal niches and vascular niches^7^,^8^,^9^,^10^. The Notch signaling pathway plays a crucial role in regulating multiple aspects of hematopoiesis during embryonic and postnatal development by mediating the HSPC-stroma interaction. In mammals, there are five Notch ligands (Delta-like [Dll] 1, 3, and 4 and Jagged 1 and 2) and four receptors (Notch 1–4). The Notch ligand-receptor interaction mediated by the Delta-Serrate-Lag-2 (DSL) domain of the ligands triggers proteolytic cleavages of the receptors, resulting in the release of Notch intracellular domain (NICD) into the cytoplasm. NICD then translocates into the nucleus and associates with a DNA-binding protein, the recombination signal-binding protein Jκ (RBP-J), and subsequently transactivates downstream genes such as the Hairy and Enhancer of Split (Hes) family members^11^.

In the hematopoietic system, Notch receptors and ligands are expressed in both the BM stromal and hematopoietic cells. Notch signaling is essential for the segregation of hematopoietic stem cells (HSCs) during embryonic definitive hematopoiesis but appears to be dispensable for the self-renewal of adult HSCs^12^,^13^. However, it has been shown that activating Notch signaling facilitates HSPC expansion *ex vivo*^14^,^15^,^16^,^17^. Transplantation of human CD34^+^ cells expanded with immobilized Dll1 has been shown to significantly reduce the average time of neutropenia in a phase I clinical trial^15^. HSPCs reside in a perivascular niche, in which endothelial cells (ECs) maintain hematopoietic homeostasis by juxtacrine and paracrine factors^8^,^18^. From these findings, we have developed a fusion protein, D1R, which is composed of the DSL domain of Dll1 and an arginine-glycine-aspartic acid (RGD) motif recognizing the endothelial integrin αVβ3 and triggering ligand endocytosis^19^. We have demonstrated that mouse D1R (mD1R) promotes HSPC expansion *ex vivo* and engraftment *in vivo* after transplantation^19^. However, whether and how intrinsic Notch signaling participates in hematopoietic recovery after irradiation has not been clearly elucidated. In this study, we address this question by using a conditional knockout of RBP-J in hematopoietic cells. Our data demonstrated that Notch signaling is critically involved in hematopoietic recovery after irradiation. The *in vivo* administration of mD1R significantly accelerated hematopoietic recovery after irradiation and treatment with cyclophosphamide (CTX). We identified colony stimulating factor 2 receptor beta 2 (Csf2rb2) as a new downstream molecule of Notch signaling, and mD1R enhanced Csf2rb2 expression in hematopoietic cells. These results suggest that the systemic administration of mD1R may have therapeutic potential to accelerate hematopoietic recovery in patients undergoing radiotherapy and chemotherapy.

## Results

### Blocking Notch signaling by conditional RBP-J knockout in the BM aggravates TBI-induced mortality and myelo-suppression in mice

**Figure 1:**
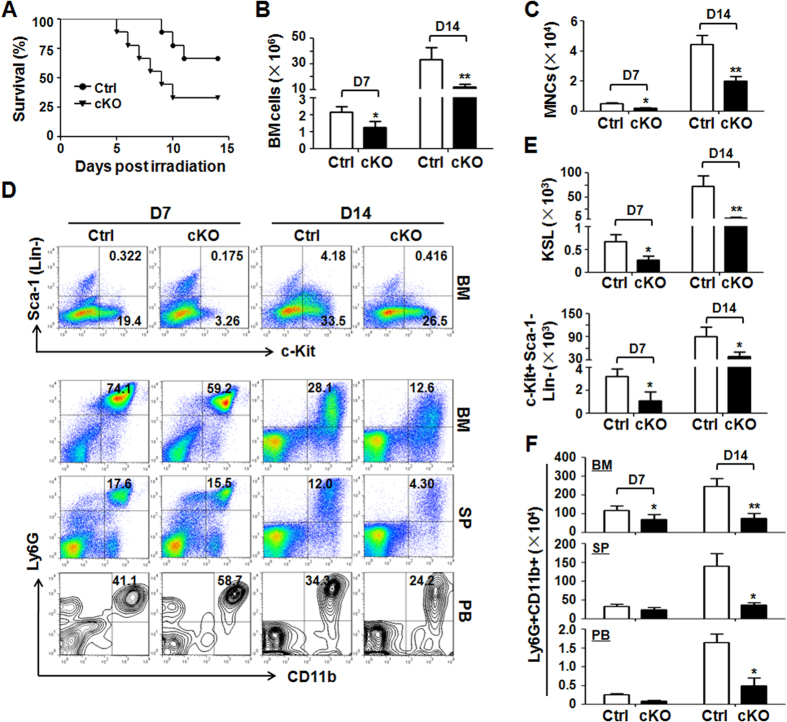
RBP-J disruption led to increased sensitivity to TBI-induced mortality and myelo-suppression. **(A)** RBP-J cKO and control mice (MxCre-RBP-J^f/f^ and MxCre-RBP-J^f/+^ mice induced with poly(I)-poly(C)) were subjected to TBI with 600 cGy of γ-radiation. The survival of mice was plotted for 14 days. **(B)** The number of nucleated BM cells was counted and compared between RBP-J cKO and control mice after TBI. **(C)** The number of MNCs in peripheral blood was counted and compared between RBP-J cKO and control mice after TBI. **(D)** FACS analysis of hematopoietic cells from RBP-J cKO and control mice after TBI. **(E)** The numbers of KSL (c-Kit^+^Sca-1^+^Lin^−^) and myeloid progenitors (c-Kit^+^Sca-1^−^Lin^−^) were compared between RBP-J cKO and control mice. **(F)** The number of Ly6G^+^CD11b^+^ monocytes in the BM, spleen (SP) and peripheral blood (PB) was compared between RBP-J cKO and control mice. Bars = means ± SD (n = 6). *P < 0.05; **P < 0.01.

To determine the role of canonical Notch signaling in TBI-induced BM damage, we generated MxCre-RBP-J^f/f^ and MxCre-RBP-J^f/+^ mice and induced homozygous (RBP-J cKO) and heterozygous (control) RBP-J disruption by the injection of poly(I)-poly(C)^20^. After TBI with 600 cGy of γ-radiation, RBP-J cKO mice exhibited reduced survival compared with the control mice (P < 0.05) (Fig. 1A). The total BM cell number and number of mononuclear cells (MNCs) in peripheral blood decreased significantly in RBP-J cKO mice (Fig. 1B,C). The analysis of cell populations showed that KSL and c-Kit^+^Sca-1^−^Lin^−^ myeloid progenitor populations^21^ in the BM decreased markedly in RBP-J cKO mice after irradiation (Fig. 1D,E). Ly6G^+^CD11b^+^ monocytes also declined in the BM, spleen and peripheral blood of RBP-J cKO mice after TBI (Fig. 1D,F). These results suggested that blocking Notch signaling in the BM aggravated TBI-induced mortality accompanied by reduced BM cells in mice.

### Activation of Notch with mD1R improves BM recovery and myelogenesis after TBI

**Figure 2:**
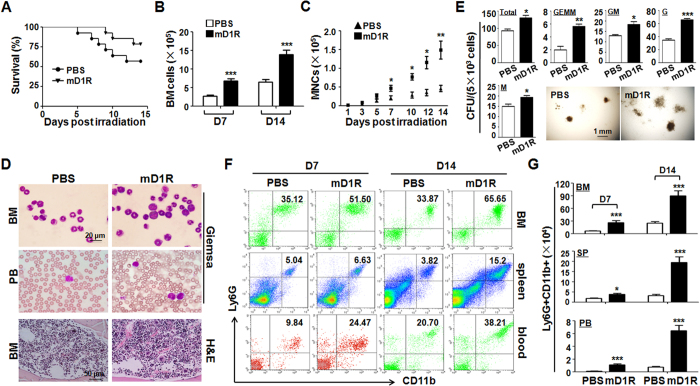
Activation of Notch signaling by mD1R improved radiation tolerance and accelerated myeloid recovery. **(A)** Eight-week-old C57BL/6 mice subjected to sublethal TBI were injected i.p. with mD1R (4 mg/kg) or PBS every day for 14 days. The survival of the mice was plotted. **(B,C)** The total numbers of nucleated cells in the BM (**B**) and peripheral blood (**C**) were determined every two days. **(D)** Cells from the BM and peripheral blood (PB) were stained with Giemsa-Wright staining. Bottom, femurs were subjected to H&E staining. **(E)** BM cells from mice on day 7 in (**A**) were subjected to colony-forming assays. The numbers of total CFU, GEMM-CFU, GM-CFU, G-CFU, and M-CFU were compared. The inset pictures show typical colonies in the two groups (n = 4). **(F**,**G)** Single-cell suspensions were prepared from the BM, spleen (SP) and peripheral blood (PB) of the mice in (**A**) on day 7 and day 14 and were analyzed by FACS (**F**). The numbers of Ly6G^+^CD11b^+^ cells in the BM, SP and PB were compared (**G**). Bars = means ± SD (n = 6). *P < 0.05; **P < 0.01; ***P < 0.001.

Recombinant mD1R was expressed in *E. coli* and purified^19^. To determine the effect of mD1R on hematopoiesis after irradiation, mice were subjected to 600-cGy TBI and then to repeated injection with mD1R (4 mg/kg/injection). We examined the activation of Notch signaling in hematopoietic cells by using NICD immunostaining, and found that mD1R efficiently increased nuclear NICD, suggesting the activation of Notch signaling in hematopoietic cells after D1R administration (Supplementary Fig. S1). On the 7th and 14th days after irradiation, mD1R-treated mice showed increased survival (P < 0.05) (Fig. 2A), with an increased number of BM cells and peripheral blood MNCs, compared with mice injected with PBS (Fig. 2B,C). Giemsa staining and H&E staining on day 7 after the irradiation showed that mD1R efficiently promoted BM recovery after TBI (Fig. 2D).

We evaluated the effect of mD1R on the recovery of myeloid cells after irradiation. A colony-forming assay using BM cells showed that the mD1R-treated group generated a larger number of myeloid CFUs with greater sizes, including GEMM-, GM-, G- and M-CFU colonies, thus suggesting increased myeloid progenitor cells (Fig. 2E). FACS analysis indicated that the number of Ly6G^+^CD11b^+^ monocytes in the BM, spleen and peripheral blood was also reconstituted more quickly in mD1R-treated mice than in control mice after irradiation (Fig. 2F,G). These results suggested that the administration of mD1R accelerates BM recovery, particularly myeloid reconstitution after radiation injury.

### Activation of Notch signaling by mD1R protects HSPCs after TBI

**Figure 3:**
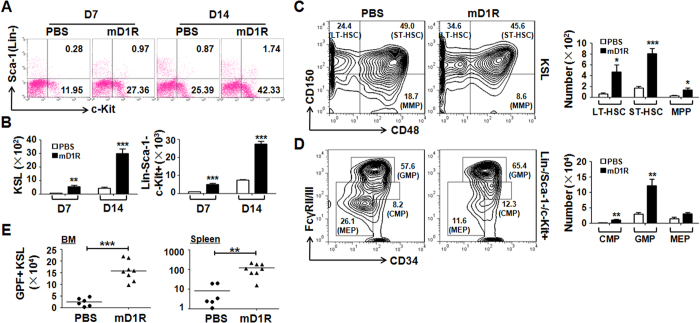
Activation of Notch signaling by mD1R protected HSPCs after TBI. **(A,B)** Eight-week-old C57BL/6 mice subjected to sublethal TBI were injected i.p. with mD1R (4 mg/kg) or PBS every day for 7 or 14 days. Single-cell suspensions were prepared from the BM and analyzed by FACS (**A**). The numbers of KSL cells (**B**, left) and c-Kit^+^Sca-1^−^Lin^−^ progenitor cells (**B**, right) were compared. **(C,D)** BM cells from mice in (**A**, D14) were subjected to FACS analyses. The numbers of LT-HSC (CD150^+^CD48^-^ KSL), ST-HSC (CD150^+^CD48^+^KSL), MPP (CD150^-^CD48^+^KSL), GMP (Lin^−^Sca-1^−^c-Kit^+^CD34^+^FcγRII/III^high^), CMP (Lin^−^Sca-1^−^c-Kit^+^CD34^+^FcγRII/III^low^), and MEP (Lin^−^Sca-1^−^c-Kit^+^CD34^-^FcγRII/III^low^) were determined and compared. **(E)** GFP^+^C57BL/6 mice were subjected to sublethal TBI and treated with mD1R or PBS for 7 days. BM cells were isolated, mixed with equivalent numbers of normal BM cells, and transplanted into lethally irradiated (900 cGy) congenic mice. The numbers of GFP^+^ KSL cells in the BM and spleen (SP) of the recipient mice were determined with FACS 8 weeks after BM transplantation. Bars = means ± SD (n = 6). *P < 0.05; **P < 0.01; ***P < 0.001.

We assessed the effect of mD1R-mediated Notch activation on HSPCs after irradiation. Mice were irradiated and treated with mD1R as described above. Flow cytometry showed that mD1R markedly increased the KSL population in the BM (Fig. 3A,B). Moreover, mD1R treatment significantly accelerated the recovery of the c-Kit^+^Sca-1^−^Lin^−^ myeloid progenitor population in the BM after irradiation (Fig. 3A,B).

We further examined the effects of mD1R-mediated Notch activation on different populations of HSPCs after irradiation. In the KSL compartment, staining with CD150 and CD48 further delineated long-term (LT)-HSCs (CD150^+^CD48^−^), short-term (ST)-HSCs (CD150^+^CD48^+^) and multipotent progenitors (MPPs) (CD150^−^CD48^+^)^22^,^23^. We found that treatment with mD1R after irradiation significantly increased the number of all of these HSPC populations in the BM (Fig. 3C). Moreover, in the c-Kit^+^Sca-1^−^Lin^−^ myeloid progenitor compartment in the BM, treatment with mD1R increased the numbers of common myeloid progenitors (CMP, CD34^+^FcγRII/III^low^) and granulocyte/monocyte progenitors (GMP, CD34^+^FcγRII/III^high^), but the number of myeloid/erythroid progenitors (MEP, CD34^−^FcγRII/III^low^) remained unchanged (Fig. 3D)^23^. These data suggested that mD1R-mediated Notch activation protected HSPCs after irradiation.

mD1R-mediated Notch activation was dose dependent (Supplementary Fig. S2A). It appeared that hematopoietic protection by mD1R after irradiation was also dose dependent in the BM and peripheral blood (Supplementary Fig. S2B–D). To functionally evaluate the protection of HSPCs by mD1R treatment after irradiation, GFP transgenic mice were irradiated with 600-cGy TBI and treated with mD1R or PBS for 7 days as described above. BM cells were collected from mD1R- or PBS-treated mice, mixed with equivalent numbers of normal BM cells, and transplanted into lethally irradiated congenic mice. The results indicated that mice that accepted BM cells from mD1R-treated mice showed an increased level of donor-cell engraftment in the BM, spleen and peripheral blood (Fig. 3E, Supplementary Fig. S3). These results suggested that mD1R treatment significantly promoted the recovery of BM HSPCs after TBI.

### Activation of Notch with mD1R improves hematopoiesis after chemotherapy in mice

**Figure 4:**
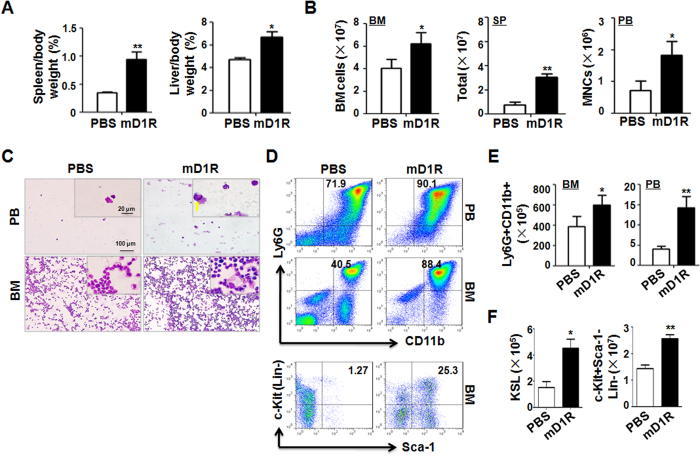
Activation of Notch signaling by mD1R protected against hematopoiesis in mice after chemotherapy. **(A)** C57BL/6 mice were injected i.p. once with CTX (150 mg/kg), followed by PBS or mD1R (4 mg/kg) every day for 7 days. The ratios of spleen weight/body weight (left) and liver weight/body weight (right) were measured. **(B)** The numbers of nucleated cells in the BM (left), spleen (SP, middle), and peripheral blood MNCs (PB, right) were determined. **(C)** Peripheral blood (PB) and BM were collected and subjected to Giemsa and H&E staining, respectively. **(D–F)** Single-cell suspensions were prepared from the BM and peripheral blood (PB) of mice in (**A**) and were analyzed by FACS (**D**). Monocytes (Ly6G^+^CD11b^+^) in the BM and peripheral blood were calculated and compared (**E**). In (**F**), the numbers of KSL cells and c-Kit^+^Sca-1^−^Lin^−^ progenitor cells were compared. Bars = means ± SD (n = 6). *P < 0.05; **P < 0.01.

Chemotherapy frequently results in BM suppression. We examined the effects of mD1R on chemotherapy-induced BM suppression. Mice were challenged with a single dose of CTX. mD1R or PBS was injected daily from the day following CTX challenge. The results showed that treatment with mD1R significantly increased the spleen/body and liver/body weight ratios (Fig. 4A). The total numbers of BM cells, spleen cells and peripheral blood MNCs also increased in mice treated with mD1R (Fig. 4B), and results were confirmed by Wright-Giemsa staining and H&E staining of peripheral blood and BM (Fig. 4C), respectively. Flow cytometry analysis showed that mD1R treatment significantly increased Ly6G^+^CD11b^+^ monocytes in the BM and peripheral blood (Fig. 4D,E). The numbers of KSL and c-Kit^+^Sca-1^−^Lin^−^ progenitors also increased in mD1R-treated mice (Fig. 4D,F). These data suggested that mD1R improved chemotherapy-induced BM suppression.

### mD1R promotes the proliferation and survival of myeloid cells in the BM after irradiation

G-CSF is the major cytokine promoting granulopoiesis^24^,^25^. We evaluated whether mD1R could synergize with G-CSF in promoting hematopoietic recovery after irradiation. Mice were irradiated with 600-cGy TBI, and this was followed by i.p. injection with PBS, G-CSF, mD1R, or mD1R plus G-CSF for 7 days. Analyses of BM and peripheral blood cells showed that, whereas mD1R or G-CSF treatment markedly elevated hematopoiesis after irradiation, the combined application of mD1R and G-CSF did not show additive effects on hematopoiesis (Supplementary Fig. S4A–G), thus suggesting that mD1R and G-CSF promote HSPCs and myeloid recovery after irradiation but probably through distinct mechanisms.

**Figure 5:**
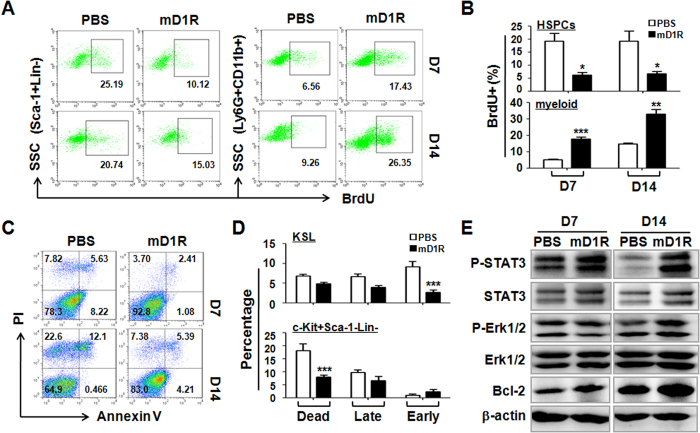
Notch signaling regulated the proliferation and apoptosis of hematopoietic cells after irradiation *in vivo*. **(A,B)** C57BL/6 mice subjected to sublethal TBI were injected i.p. with mD1R (4 mg/kg) or PBS every day for 14 days. Meanwhile, the mice were injected i.p. with BrdU (100 mg/kg) every two days and were maintained with drinking water containing BrdU (1 mg/ml) until the analysis of BM cells by FACS (**A**). The percentages of BrdU^+^ HSPCs (Sca-1^+^Lin^−^) and myeloid cells (Ly6G^+^CD11b^+^) in the BM were compared (**B**). **(C**,**D)** KSL cells in the BM from mice in (**A**) were analyzed by FACS after staining with Annexin V and PI (**C**). The apoptosis of KSL and Lin^−^Sca-1^−^c-Kit^+^ cells in the BM was compared on day 7 after irradiation. **(E)** Lin^−^ cells in the BM from mice in (**A**) were sorted by FACS. The levels of P-STAT3, STAT3, P-Erk1/2, Erk1/2, and Bcl-2 were determined by using Western blotting. The gels had been run under the same experimental conditions, and cropped gels are presented to show the targeted bands. Bars = means ± SD (n = 6). *P < 0.05; **P < 0.01; ***P < 0.001.

To access the mechanisms through which mD1R protected BM HSPCs and myeloid cells from irradiation, we evaluated cell proliferation in the BM of the irradiated mice. Irradiated mice were treated with mD1R as described above, and proliferating cells were labeled with BrdU. FACS analysis was used to detect BrdU-positive HSPCs (Sca-1^+^Lin^−^) and monocytes (Ly6G^+^CD11b^+^). As shown in Fig. 5A,B, mD1R treatment of mice after 600-cGy TBI resulted in a decrease in BrdU^+^ HSPCs, but the number of BrdU^+^ monocytes (Ly6G^+^CD11b^+^) increased significantly. Then, we examined the apoptosis of KSL and c-Kit^+^Sca-1^−^Lin^−^ myeloid progenitors in the BM of irradiated mice treated with mD1R by using Annexin V and PI staining. The result showed that mD1R treatment significantly reduced the level of irradiation-induced early apoptosis and dead cells in both of the KSL and c-Kit^+^Sca-1^−^Lin^−^ populations (Fig. 5C,D). We assessed the activation of a few signaling pathways that are potentially involved in cell proliferation and survival using western blot analysis of Lin^−^ BM cells from irradiated mice treated with mD1R^26^,^27^. The results showed that mD1R increased the phosphorylation of STAT3 and ERK1/2 (Fig. 5E). Moreover, the expression of the anti-apoptotic protein Bcl-2 was upregulated upon mD1R treatment after TBI (Fig. 5E). These data suggested that mD1R treatment attenuated the proliferation of HSPCs and increased the proliferation of myeloid cells on the one hand, and reduced the apoptosis of HSPCs and myeloid progenitors on the other hand, in the BM after irradiation.

### Csf2rb2 is a downstream molecule of Notch signaling in BM cells

**Figure 6:**
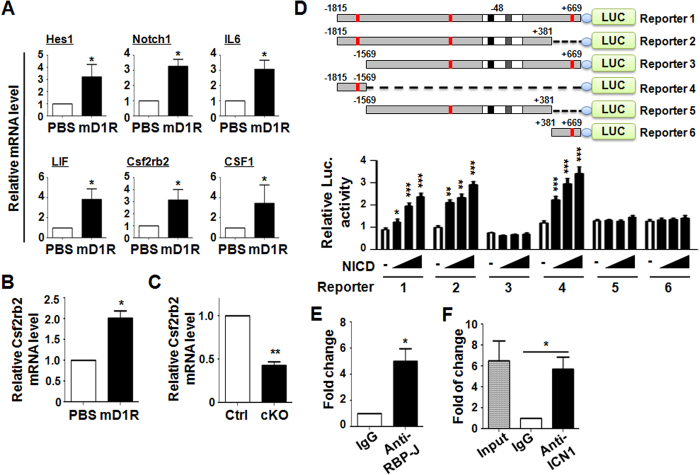
Csf2rb2 was downstream of Notch signaling. **(A)** Lin^−^ cells were isolated from the mouse BM and co-cultured with HUVECs + m5GF in the presence of PBS or mD1R. Floating hematopoietic cells were collected 48 h after the start of the culture. The expression levels of Hes1, Notch1, IL-6, Lif, Csf2rb2 and Csf1 were determined by using qRT-PCR. **(B)** Mice treated with mD1R or PBS were subjected to sublethal TBI. Lin^−^ cells were isolated on day 7, and Csf2rb2 mRNA expression was determined by using qRT-PCR. **(C)** RBP-J cKO and control mice were subjected to sublethal TBI. Lin^−^ cells were isolated on day 7, and Csf2rb2 mRNA expression was determined by using qRT-PCR. **(D)** Reporter assay. Upper, schematic representation of the reporter constructs driven by the mouse Csf2rb2 gene promoter and its derivatives. Red blocks represent putative RBP-J-binding sites. Lower, luciferase assay. HeLa cells were co-transfected with increasing amounts of pEF-BOS-NICD and the reporter constructs (Reporter 1~6) as described above. Cells were lysed 24 h after transfection, and luciferase activities were evaluated. **(E**,**F)** ChIP assay. BM cells were treated with 1% formaldehyde to crosslink chromatin and subjected to immunoprecipitation using anti-RBP-J (**E**) or anti-NICD (**F**), with preimmune IgG as a control. Precipitated chromatin fragments were further analyzed by qPCR using primers spanning the most distal putative RBP-J-binding sites (Fig. 6D, upper) in the Csf2rb2 promoter. Bars = means ± SD (n = 6). *P < 0.05; **P < 0.01; ***P < 0.001.

We next sought to reveal the mechanism by which mD1R promoted HSPC and myeloid recovery after irradiation. A gene-set enrichment analysis^28^,^29^ of our previous gene expression profiling data derived from mD1R-expanded KSL cells^19^ revealed a statistically significant de-repression of an extended myeloid-specific program in the mD1R group (Supplementary Fig. S5). Among a group of candidate genes, quantitative RT-PCR showed that 6 genes, Notch1, Hes1, IL-6, Csf1, Lif and Csf2rb2^30^, were upregulated more than 3-fold in mD1R-stimulated Lin^−^ BM cells (Fig. 6A). *In vivo*, mD1R-treated mice showed higher expression, whereas RBP-J cKO mice exhibited decreased expression of Csf2rb2 in Lin^−^BM cells (Fig. 6B,C), thus suggesting that Csf2rb2 might be a target gene of Notch signaling in BM Lin−, likely hematopoietic progenitor, cells.

The Csf2rb2 promoter region contains potential RBP-J-binding sites (TGGGAA) at −1680, −426, and +602. To determine whether Notch signaling could directly transactivate the Csf2rb2 promoter, we cloned the mouse Csf2rb2 promoter fragment and examined the transactivation of the Csf2rb2 promoter by NICD using a luciferase reporter assay. As shown in Fig. 6D, co-transfection of the NICD-expressing vector induced the expression of luciferase from pGL3-CSF2RB2 in a dose-dependent manner, thus suggesting that Notch signaling transactivates the Csf2rb2 promoter. We truncated the Csf2rb2 promoter to make different reporter constructs (Fig. 6D). Reporter assays confirmed that the element responding to Notch signaling was located between −1815 and −1569 of the Csf2rb2 promoter, which contains a putative RBP-J-binding site. In line with this finding, ChIP assay revealed the direct occupancy of this fragment (−1815 to −1569) by RBP-J (Fig. 6E) and NICD (Fig. 6F). Collectively, these data suggested that Notch signaling might promote hematopoietic reconstitution through activating Csf2rb2 expression.

### Csf2rb2 is required for mD1R to promote hematopoiesis

**Figure 7:**
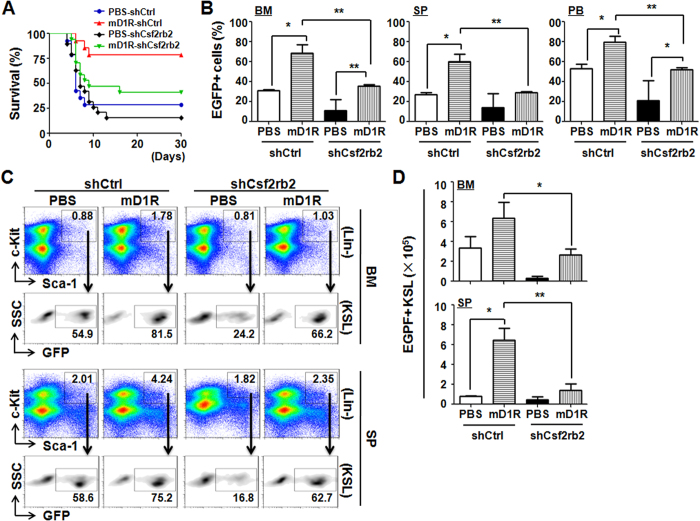
Activation of Notch signaling with mD1R protected hematopoietic cells through Csf2rb2. **(A)** C57BL/6 mice subjected to lethal TBI (900 cGy) were injected i.p. with mD1R (4 mg/kg) or PBS every other day for 30 days. At 2 h post irradiation, the mice were injected with lentiviral particles expressing Csf2rb2-shRNA-EGFP (shCsf2rb2) or control-shRNA-EGFP (shCtrl) through the caudal veins. The survival of the mice was plotted for 30 days (n = 20). **(B)** The percentage of EGFP^+^ cells in the BM, spleen (SP) or peripheral blood (PB) of the mice in (**A**) was determined and compared. **(C**,**D)** BM and spleen (SP) cells were analyzed by FACS, and EGFP^+^ KSL cells were compared. Bars = means ± SD (n = 10). *P < 0.05; **P < 0.01.

To assess whether Notch signaling affects hematopoiesis through Csf2rb2 after TBI *in vivo*, we used shRNA to disrupt Csf2rb2 expression. Three Csf2rb2-specific shRNAs were evaluated in BM KSL cells to knock down Csf2rb2. All Csf2rb2-specific shRNAs reduced the Csf2rb2 mRNA levels, and Csf2rb2-shRNA 2 was selected in further studies (Supplementary Fig. S6). Normal KSL cells were transfected with either control-EGFP or Csf2rb2-shRNA-EGFP, mixed with normal BM cells and transplanted into lethally irradiated C57BL/6 recipient mice. The recipient mice were administered 4 mg/kg mD1R or PBS every other day for 30 days. As shown in Fig. 7A,B, mD1R treatment efficiently improved the survival and hematopoietic reconstitution after KSL^+^ BM cell transplantation in lethally irradiated mice compared with PBS-treated recipients. However, the mD1R-mediated hematopoietic reconstitution was significantly impaired by the transfection of KSL cells with Csf2rb2 shRNA (Fig. 7A,B). In the KSL compartments in the BM and spleen, Csf2rb2-shRNA-transfected cells (EGFP^+^) showed a reduced percentage of chimerism (Fig. 7C,D). These data suggested that mD1R-mediated hematopoietic reconstitution was at least partially dependent on the activation of Csf2rb2.

## Discussion

Genotoxic stresses, such as those derived from radio- and chemotherapy, preferentially damage BM HSPCs^31^,^32^,^33^. Although Notch signaling acts as an important regulator of BM HSPCs by controlling self-renewal and affecting lineage fates in development and homeostasis^34^,^35^, the role of Notch signaling in stress-induced BM damage has not been fully revealed. In this study, using an inducible RBP-J knockout mouse model, we showed that the disruption of Notch signaling in the BM aggravated radiation-induced BM damage. However, because the activation of Notch signaling in HSCs results in massive T-cell differentiation in the BM, we used a recombinant soluble mDll1 ligand fused with an EC-targeting RGD peptide to activate Notch in BM HSPCs after BM damage. Systemic treatment with mD1R after sublethal TBI or chemotherapy decreased the mortality and supported the repopulating capacity of HSPCs in mice and markedly enhanced hematopoietic recovery. In agreement with this finding, the number of KSL cells in the BM and spleen was markedly elevated during BM recovery. These results indicated that the activation of Notch signaling protects BM HSPCs from damage induced by genotoxic stresses, such as radiotherapy and chemotherapy.

Notably, treatment with mD1R resulted in a significant improvement in myeloid reconstitution, thus revealing a specific effect on short-term HSPCs with myeloid repopulating potential. It has been previously shown that Notch signaling impedes myeloid differentiation^36^. Nevertheless, Delaney *et al.* have reported that patients who receive transplantation of cord blood progenitors expanded with immobilized Delta1^ext-IgG^ achieve early and rapid myeloid engraftment^15^. Recent studies have also suggested that Notch activation promotes myeloid differentiation^37^. Therefore, the activation of Notch signaling with mD1R protein not only promotes HSPC recovery after BM damage but also enhances myelogenesis, which might be important to protect against infections that are frequently lethal to patients receiving radio- and chemotherapy.

It is likely that the Notch pathway facilitates BM recovery and myelogenesis after BM damage via several mechanisms^33^, such as the alteration of Cebpα expression in primitive myeloid cells^13^, regulation of fucosylation in myelopoiesis^34^, or control of IL-6-JAK-STAT signaling in myeloid progenitor cells^26^. The angiocrine factors derived from endothelial cells in the BM niche, which is regulated by Notch signaling, might also play important roles^38^. Our results showed that mD1R increased the phosphorylation of STAT3 and Erk1/2 and upregulated the expression of Bcl-2. These changes might be might be directly induced upon triggering of Notch signaling, and might also be indirectly induced by signals from other mD1R-stimulated cells, such as endothelial cells. Our data identified a novel mechanism of BM recovery after the activation of Notch signaling by mD1R, namely the elevated expression of Csf2rb2, a common β subunit (βc) of granulocyte-macrophage colony-stimulating factor (GM-CSF), interleukin-3 (IL-3) and IL-5^39^,^40^. As the major signaling mediator of the βc cytokine receptor complex, Csf2rb2 confers hematopoietic survival and promotes myeloid differentiation at very low concentrations by activating downstream signaling pathways such as the JAK2/STAT5, Ras/Raf/MAPK and PI3-kinase/Akt pathways^41^,^42^,^43^. In contrast to those of many other cytokines, the biological activities of βc cytokines are largely dispensable for the maintenance of steady-state functions. Moreover, by offering docking sites for many signaling molecules, the βc is the principal signal transducing subunit regulating cell survival, proliferation and differentiation in health and diseases^42^,^43^. Therefore, it is possible that Notch-induced Csf2rb2 contributes to the reduced cell death in HSPCs and the increased myelogenesis after radiation. However, further investigation is required to sufficiently elucidate the role of Csf2rb2 and its relationship with Notch signaling in hematopoiesis.

G-CSF is currently used for the treatment of BM failure from unanticipated radiation exposure or after transplantation^24^,^25^. We found no additive effect of mD1R and G-CSF on myeloid reconstitution, possibly because of their different mechanisms: G-CSF modulates neutrophils by triggering myeloid progenitor proliferation and differentiation^43^, whereas D1R mediates the early recovery of the hematopoietic progenitor pool after irradiation^44^. In addition, mD1R stimulates ECs and is likely to alter the spectrum of angiocrine factors secreted by ECs. These angiocrine factors have been demonstrated to play important roles in regulating hematopoiesis. Further studies are required to elucidate the effects of Notch signaling in ECs on the secretion of angiocrine factors during stress-stimulated hematopoiesis.

Current clinical interventions to mitigate radiation toxicity have relied on a combination of supportive care, growth factors, cytokines, and specific chelating agents^45^,^46^. In the current study, we demonstrated that Notch signaling promotes HSPC regeneration after radiation-induced BM damage. Importantly, mD1R ameliorates DNA damage-induced myelo-suppression. Although late hematological toxicity has been associated with growth factor support after exposure to DNA-damaging agents in both humans and mice^47^, mD1R appears not to augment late hematological toxicity after TBI. Furthermore, we elucidated a novel mechanism in which mD1R regulates the reconstitution capacity of HSPCs via the expression of Csf2rb2. Considering the safety, availability and targeted efficacy, the systemic administration of mD1R may have therapeutic potential to accelerate hematopoietic recovery in patients undergoing TBI and in victims of acute radiation sickness.

## Materials and Methods

### Mice

C57BL/6 mice were maintained under specific-pathogen-free (SPF) conditions. Male mice aged 8 to 10 weeks were subjected to sublethal (600 cGy) total body irradiation (TBI) with γ-radiation from a ^60^Co irradiator. The mice were injected intraperitoneally (i.p.) with mD1R (4 mg/kg)^19^ or phosphate-buffered saline (PBS) 2 h post irradiation, and this was followed by daily injection of the same reagent for 7 or 14 days. In some experiments, male mice were injected i.p. with CTX (150 mg/kg) (Sigma-Aldrich, St Louis, MO), followed by treatment with mD1R or PBS as above. RBP-J-floxed (RBP-J^f^) mice were described previously^20^. For the induction of RBP-J deletion, RBP-J^f/f^ mice were mated with Mx-Cre mice, and mice with suitable genotypes were injected with poly(I)-poly(C) (Sigma-Aldrich) as described previously^20^. To monitor cell proliferation, the mice were injected i.p. with 5-bromo-2-deoxyuridine (BrdU; Sigma-Aldrich) (100 mg/kg in PBS) every two days after irradiation and were maintained with drinking water containing 1 mg/ml BrdU for 14 days. Granulocyte colony-stimulating factor (G-CSF) was administered i.p. at a dose of 50 μg/kg. All animal experiments were approved by and performed in accordance with guidelines from the Animal Experiment Administration Committee of the Fourth Military Medical University.

### Cell culture and transfection

HeLa cells were maintained in Dulbecco’s modified Eagle’s medium (DMEM) (Invitrogen, Carlsbad, CA) supplemented with 10% fetal bovine serum (FBS), 100 IU/ml penicillin, and 100 μg/ml streptomycin sulfate. The 5′ flanking sequence (−1815 ~ +669) of the mouse Csf2rb2 gene was amplified by PCR from mouse genomic DNA and inserted into the *Nhe* I and *Xho* I sites of the pGL3-basic vector (Promega, Madison, WI) to construct pGL3-CSF2RB2. Different truncated reporter constructs were also generated by using PCR (Fig. 5C). Transient transfection of HeLa cells was performed by using Lipofectamine 2000^TM^ (Invitrogen) according to the manufacturer’s instructions. Cells (2 × 10^4^) were seeded in 24-well plates for 24 h and transfected with 0.3 μg of reporter constructs, different amounts of pEF-BOS-NICD^48^ and 5 ng of phRL-TK. Luciferase activity was assessed 24 h after the transfection by using Luminoskan Ascent (Labsystems, Helsinki, Finland) and a Dual-Luciferase Reporter Assay Kit (Promega) according to the manufacturer’s protocol.

shRNAs targeting Csf2rb2 were inserted into pGV118-U6-shRNA-Ubi-EGFP for lentivirus packaging. c-Kit^+^Sca-1^+^Lin^−^ (KSL) cells were sorted and cultured in 48-well plates (1 × 10^3^ cells per well) with serum-free medium (StemSpan SFEM; STEM CELL Technologies, Vancouver, Canada) supplemented with thrombopoietin (TPO, 20 ng/mL), stem cell factor (SCF, 125 ng/mL), Flt-3 ligand (Flt-3L, 50 ng/mL), interleukin (IL)-6 (25 ng/mL) and IL-3 (10 ng/mL) (PeproTech, Rocky Hill, NJ). Cells were transduced with lentivirus^49^ and cultured further for 6 days. Next, the lentivirus-transfected KSL cells (5 × 10^3^) were mixed with normal BM cells (5 × 10^5^) and transplanted into lethally irradiated mice (1000 cGy) through the tail veins. Hematopoiesis in recipients was analyzed 8 weeks after the transplantation.

### Colony-forming unit (CFU) assay

The CFU assay was performed by mixing freshly isolated nucleated cells with Methocult GF M3434 medium (STEM CELL Technologies). Cells were cultured for 14 days, and colonies (with more than 50 cells) containing different lineages of cells were counted under a microscope.

### Flow cytometry

Fluorescence-activated cell sorting (FACS) analysis was performed routinely by using a FACSCalibur^TM^ and FACSAriaII flow cytometer (BD Immunocytometry Systems). Data were analyzed with the FlowJo vX.0.6 software (FlowJo, LLC, Ashland, OR). The antibodies used in the analyses are listed in Supplementary Table S1. Dead cells were excluded by using propidium iodide (PI) staining. Apoptosis was analyzed by staining using an Annexin V Apoptosis Detection Kit (eBioscience, San Diego, CA), followed by FACS analysis.

### Real-time reverse transcription (RT)-polymerase chain reaction (PCR)

Total RNA was extracted with TRIzol reagent (Invitrogen). cDNA was prepared by using a reverse transcription system (Takara, Dalian, China). Quantitative real-time PCR was performed in triplicate by using a kit (SYBR Premix EX Taq, Takara) and the ABI Prism 7500 Real-Time PCR System, with β-actin as an internal control. The primers are listed in Supplementary Table S2.

### Western blot analysis

Western blot analysis was performed routinely with primary antibodies against Erk1/2, pErk1/2, STAT3, pSTAT3, Bax, Bcl-2, and β-actin. Horseradish peroxidase (HRP)-conjugated goat anti-rabbit IgG and goat anti-mouse IgG were used as secondary antibodies.

### Histology

Tissues were fixed in 4% paraformaldehyde overnight at 4 °C. Femurs were decalcified for 20 days using an EDTA decalcifying solution for paraffin sections. Hematoxylin and eosin (H&E) staining was performed according to routine protocols. The BM and peripheral blood smears were stained with Wright-Giemsa stain (Sigma-Aldrich) for 10 min, and then rinsed in distilled water for 3 min. Immunofluorescence staining of cells was conducted according to standard protocols using rabbit anti-NICD1, followed by Cy3-conjugated goat anti-rabbit IgG. Images were acquired under a fluorescence microscope (BX51, Olympus, Tokyo, Japan) or a confocal microscope (FV1000, Olympus). Pixels for each color were quantified with Image Pro Plus 6.0 software to quantitatively represent the intensity of positive cells.

### Chromatin immunoprecipitation (ChIP)

BM cells (5 × 10^6^) were cross-linked with 1% formaldehyde at room temperature for 10 min. After homogenization, nuclei were collected and sonicated on ice to obtain fragmented chromatin with an average DNA length of 0.5 kb. The samples were centrifuged and pre-cleared with protein G beads in the presence of sonicated λDNA and bovine serum albumin (BSA) for 2 h at 4 °C. The supernatant was immunoprecipitated with an anti-RBP-J antibody, and the immune complexes were collected by using protein G-Sepharose beads, washed, eluted from the beads and incubated for 5 h at 65 °C to reverse the cross-links. After proteinase K treatment, DNA was extracted with phenol-chloroform and precipitated with ethanol, and this was followed by quantitative real-time PCR using the primers listed in Supplementary Table S2.

### Statistics

Statistical analyses were performed with the SPSS 12.0 program. Survival was analyzed by using Kaplan-Meier analysis. Comparisons between groups were performed by using unpaired Student’s t tests. The results are expressed as the mean ± SD. P < 0.05 was considered to be significant.

## Additional Information

**How to cite this article**: Chen, J.-J. *et al.* Disruption of Notch signaling aggravates irradiation-induced bone marrow injury, which is ameliorated by a soluble Dll1 ligand through Csf2rb2 upregulation. *Sci. Rep.* **6**, 26003; doi: 10.1038/srep26003 (2016).

## Supplementary Material

Supplementary Information
