# Supplementary material for: Disruption of Notch signaling aggravates irradiation-induced bone marrow injury, which is ameliorated by a soluble Dll1 ligand through Csf2rb2 upregulation
Source: Sci Rep. 2016 May 18;6:26003. doi: 10.1038/srep26003 (PMC4870557; doi:10.1038/srep26003)
Supplement: Supplementary Information [file srep26003-s1.doc]

**Disruption of Notch signaling aggravates irradiation-induced bone marrow injury, which is ameliorated by a soluble Dll1 ligand through Csf2rb2 upregulation**

Juan-Juan Chen, Xiao-Tong Gao, Lan Yang, Wei Fu, Liang Liang, Jun-Chang Li, Bin Hu, Zhi-Jian Sun, Si-Yong Huang, Yi-Zhe Zhang, Ying-Min Liang, Hong-Yan Qin, Hua Han

**Supplementary materials**

**Supplementary Table S1. Antibodies used in this study.**

| **Name** | **Supplier** | **Clone #** |
| --- | --- | --- |
| anti-mouse BrdU-APC | eBioscience | Bu20A |
| anti-mouse CD34-percp/cy5.5 | Biolegend | HM34 |
| anti-mouse FcγRII/III-FITC | BD Pharmingen | 2.4G2 |
| anti-mouse CD150-percp/cy5.5 | Biolegend | TC15-12F12.2 |
| anti-mouse CD48-PE | Biolegend | HM48-1 |
| anti-mouse CD3ε-APC | Biolegend | 145-2C11 |
| anti-mouse B220-APC | Biolegend | RA3-6B2 |
| anti-mouse Ly6G-FITC | BD Pharmingen | 1A8 |
| anti-mouse CD11b-APC | Biolegend | M1/70 |
| anti-mouse Sca-1-FITC | eBioscience | D7 |
| anti-mouse c-Kit-PE | Biolegend | 2B8 |
| Cy3-AffiniPure Goat Anti-Rabbit IgG (H+L) | Jackson Immuno | polyclonal |
| Rabbit polyclonal to activated Notch1 | Abcam | polyclonal |
| anti-mouse Streptavidin-APC | Biolegend | polyclonal |
| anti-RBP-J | Santa Cruz | polyclonal |
| APC-conjugated anti-mouse lineage antibody cocktail | BD Pharmingen | 145-2C11,M1/70,RA3-6B2,  TER-119, Ly-76, RB6-8C5 |
| rabbit anti-NICD1 | Abcam | polyclonal |
| Cy3-conjugated goat anti-rabbit IgG | Jackson | polyclonal |
| Erk1/2 | Cell Signaling | 137F5 |
| pErk1/2 | Cell Signaling | D13.14.4E |
| STAT3 | Cell Signaling | D3Z2G |
| pSTAT3 | Cell Signaling | D3A7 |
| Bax | Cell Signaling | Polyclonal |
| Bcl-2 | Cell Signaling | D17C4 |
| β-actin | Sigma-Aldrich | AC-15 |
| HRP-conjugated goat anti-rabbit IgG | Boster BioTec | polyclonal |
| goat anti-mouse IgG | Boster BioTec | polyclonal |

**Supplementary Table S2. Sequence of oligonucleotides and primers.**

| **Gene** | **Purpose** | **Sequence** |
| --- | --- | --- |
| Cre-F | Genotyping | 5’-CCGGTCGATGCAACGAGTGATGAGG |
| Cre-R | Genotyping | 5’-GCCTCCAGCTTGCATGATCTCCGG |
| RBP-J-F | Genotyping | 5'-GTTCTTAACCTGTTGGTCGGAACC |
| RBP-J-WT-R | Genotyping | 5'-GCTTGAGGCTTGATGTTCTGTATTGC |
| RBP-J-floxed-R | Genotyping | 5'-ACCGGTGGATGTGGAATGTGT |
| β-actin-F | RT-PCR | 5’-CATCCGTAAAGACCTCTATGCCAAC |
| β-actin-R | RT-PCR | 5’-ATGGAGCCACCGATCCACA |
| mus Hes1-F | RT-PCR | 5’-AAAGACGGCCTCTGAGCAC |
| mus Hes1-R | RT-PCR | 5’-GGTGCTTCACAGTCATTTCCA |
| mus Hes5-F | RT-PCR | 5’-CTGGAGATGGCCGTCAGCTA |
| mus Hes5-R | RT-PCR | 5’-GTAGTCCTGGTGCAGGCTCTTG |
| mus hey1-F | RT-PCR | 5’-CATGAAGAGAGCTCACCCAGA |
| mus hey1-R | RT-PCR | 5’-CGCCGAACTCAAGTTTCC |
| mus hey2-F | RT-PCR | 5’-GAGGAAACGACCTCCGAAA |
| mus hey2-R | RT-PCR | 5’-GACCTCATCACTGAGCTTGTAGC |
| mus Csf2rb2-F | RT-PCR | 5’-TTCCAGCCAGATCGTGACCT |
| mus Csf2rb2-R | RT-PCR | 5’-CCCCAAGAGATACACTCCATTCC |
| mus IL-6-F | RT-PCR | 5’-AAAGAGTTGTGCAATGGCAATTCT |
| mus IL-6-R | RT-PCR | 5’-AAGTGCATCATCGTTGTTCATACA |
| mus Csf1-F | RT-PCR | 5’-CGACATGGCTGGGCTCCC |
| mus Csf1-R | RT-PCR | 5’-CGCATGGTCTCATCTATTAT |
| mus Lif-F | RT-PCR | 5’-ATGTGCGCCTAACATGACA |
| mus Lif-R | RT-PCR | 5’-TATGCGACCATCCGATACAG |
| mus Notch1-F | RT-PCR | 5’-TGCCAGGACCGTGACAACTC |
| mus Notch1-R | RT-PCR | 5’-CACAGGCACATTCGTAGCCATC |
| mus Csf2rb2-F1 | ChIP | 5’-GCGCTAGCTTGAGTGAGAATGGATCTCTTAGGACAA |
| mus Csf2rb2-R1 | ChIP | 5’-GCCTCGAGAGAAACACATACACACATACATACACAC |
| mus Csf2rb2-F2 | ChIP | 5’-GCGCTAGCTTGAGTGAGAATGGATCTCTTAGGACAA |
| mus Csf2rb2-R2 | ChIP | 5’-GCCTCGAGGCAAAGTTAGAAGGAACACAGTAGTTAG |
| mus Csf2rb2-F3 | ChIP | 5’-GCGCTAGCGATGGTGGACATGATCATTTATGCATTG |
| mus Csf2rb2-R3 | ChIP | 5’-GCCTCGAGAGAAACACATACACACATACATACACAC |
| mus Csf2rb2-F4 | ChIP | 5’-GCGCTAGCTTGAGTGAGAATGGATCTCTTAGGACAA |
| mus Csf2rb2-R4 | ChIP | 5’-GCCTCGAGCAATGCATAAATGATCATGTCCACCATC |
| mus Csf2rb2-F5 | ChIP | 5’-GCGCTAGCGATGGTGGACATGATCATTTATGCATTG |
| mus Csf2rb2-R5 | ChIP | 5’-GCCTCGAGGCAAAGTTAGAAGGAACACAGTAGTTAG |
| mus Csf2rb2-F6 | ChIP | 5’-GCGCTAGCCTAACTACTGTGTTCCTTCTAACTTTGC |
| mus Csf2rb2-R6 | ChIP | 5’-GCCTCGAGAGAAACACATACACACATACATACACAC |
| GAPDH-F | RT-PCR | 5’-TGGCACCCAGCACAATGAA |
| GAPDH-R | RT-PCR | 5’-CTAAGTCATAGTCCGCCTAGAAGCA |
| sh-Csf2rb2 (1) | knockdown | 5’-GGTACAGGACATACAGGAA |
| sh-Csf2rb2 (2) | knockdown | 5’-TCTCCACTTTGGCCGTGTT |
| sh-Csf2rb2 (3) | knockdown | 5’-TCAGAGAGCTGGAAGGACA |


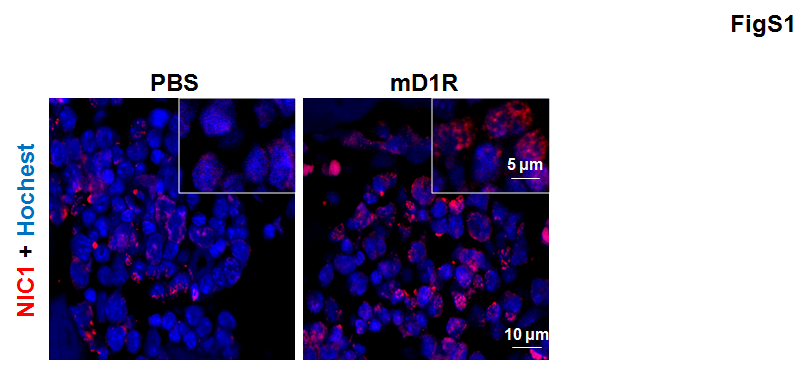


**Supplementary Figure S1. Administration of mD1R in vivo activated Notch signaling in mice.** Eight-week-old C57BL/6 mice subjected to sublethal TBI were injected i.p with mD1R (4 mg/kg) or PBS every day for 7 days. BM cells were collected from femurs and subjected to immunofluorecsence staining with anti-NIC1. Nuclei were counter-stained with Hochest.

**
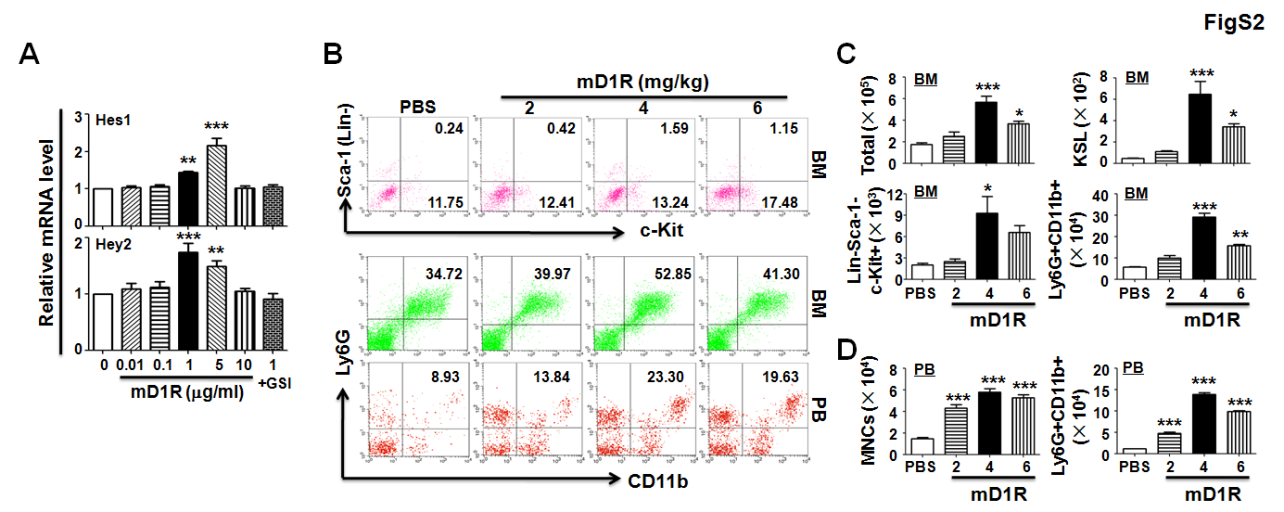
**

**Supplementary Figure S2. Protection of hematopoiesis by mD1R after irradiation was dose-dependent. (A)** bEND.3 cells were treated with different amount of mD1R. Cells were harvested and the expression of Hes1 and Hey2 was determined by qRT-PCR. **(B)** Eight-weeks-old C57BL/6 mice subjected to sublethal TBI were injected i.p with different doses of mD1R (2, 4, 6 mg/kg) or PBS every day for 7 days. BM and peripheral blood (PB) cells were analyzed by using FACS. **(C)** The total numbers of nucleated cells, KSL cells, c-Kit^+^Sca-1^-^Lin^-^ cells and CD11b^+^Ly6G^+^ cells in BM were compared. **(D)** The total numbers of MNCs and Ly6G^+^CD11b^+^ cells in PB were compared. Bars = means ± SD (n = 8). *, P < 0.05, **, P < 0.01, ***, P < 0.001.


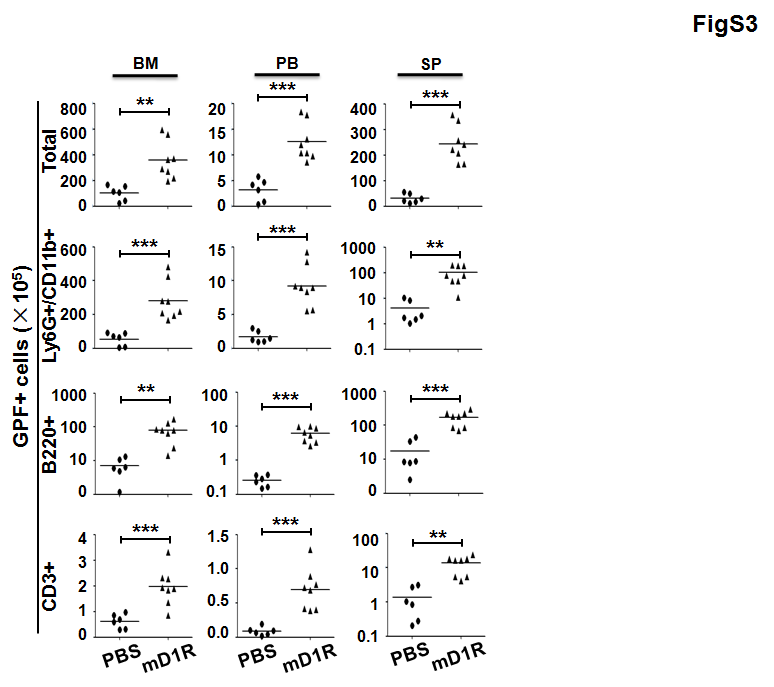


**Supplementary Figure S3. mD1R treatment protected HSPC after TBI.** C57BL/6 GFP^+^ mice were subjected to sublethal TBI and treated with mD1R or PBS for 7 days. BM cells were isolated, mixed with equivalent numbers of normal BM cells, and transplanted into lethally irradiated (900 cGy) congenic mice. The numbers of total GFP^+^ cells, GFP^+^ myeloid cells (Ly6G^+^CD11b^+^), GFP^+^ B-cells (B220^+^) and GFP^+^ T-cells (CD3^+^) engraftment in the BM, peripheral blood (PB) and spleen (SP) of the recipient mice were determined by using FACS 8 weeks after the BM transplantation. Bars = means ± SD (n = 8). *, P < 0.05, **, P < 0.01, ***, P < 0.001.


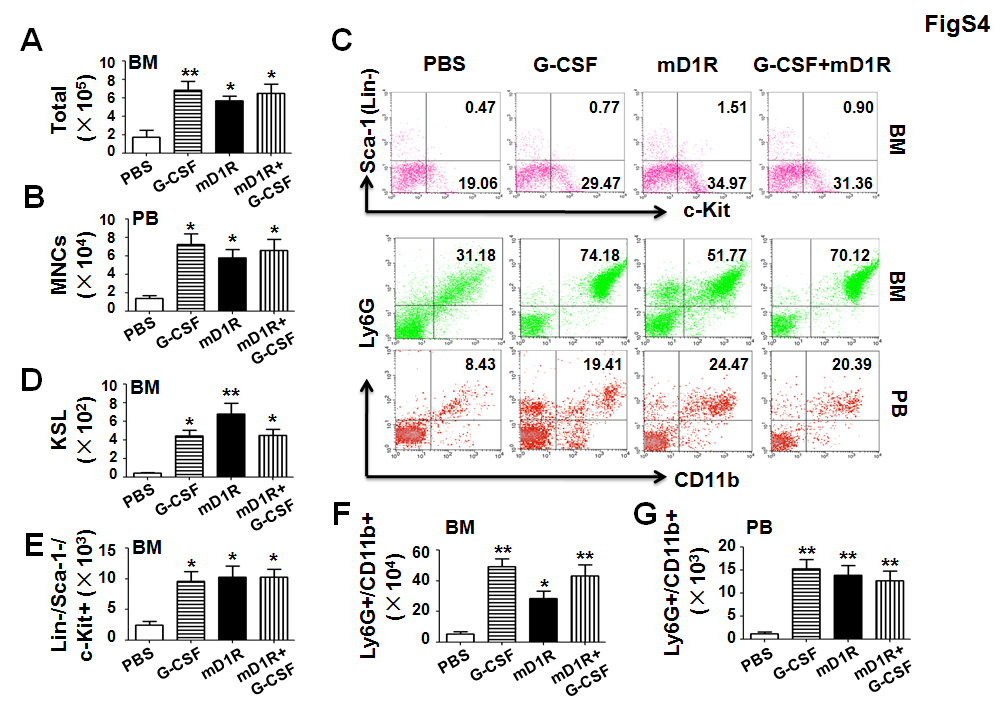


**Supplementary Figure S4. mD1R did not synergize with G-CSF in promoting hematopoiesis after irradiation. (A)** Eight-weeks-old C57BL/6 mice subjected to sublethal TBI were injected i.p with PBS, G-CSF (1 μg), mD1R, or G-CSF + mD1R every day for 7 days. BM and blood cells were analyzed by FACS. **(B, C)** The total numbers of nucleated cells in BM (B) and in peripheral blood (C) were determined on day 7 post irradiation. **(D, E)** The numbers of KSL cells (D) and Lin^-^Sca-1^-^c-Kit^+^ cells (E) in BM were calculated according to (A). **(F, G)** The numbers of Ly6G^+^CD11b^+^ cells in BM (F) and blood (G) were compared according to (A). Bars = means ± SD (n = 6). *, P < 0.05, **, P < 0.01.


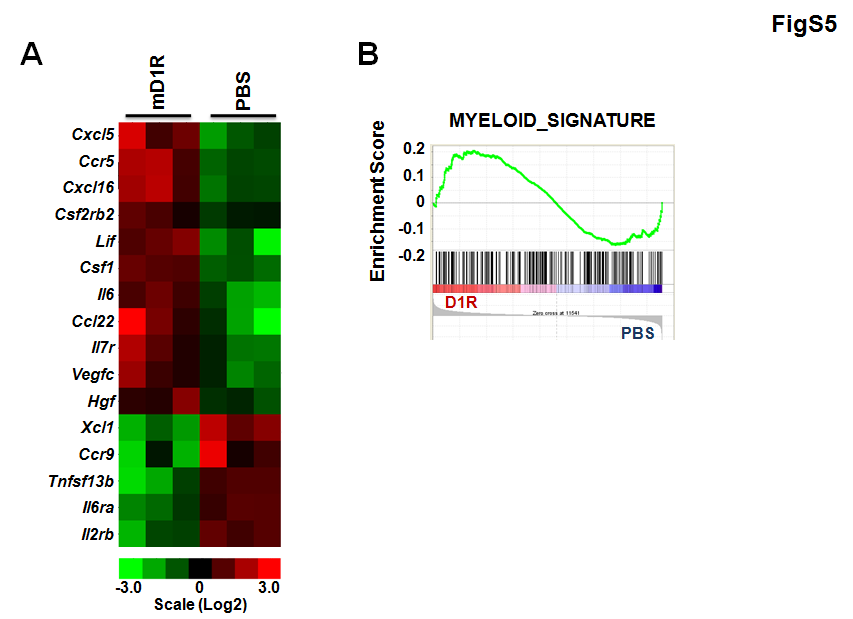


**Supplementary Figure S5. Bioinformatic analysis of gene expression profiling data of mD1R-treated BM cells.** Gene expression profiling of KSL cells treated with PBS or mD1R was described previously (Tian DM, et al. Stem Cell Res. 2013; 11:693–706). The original data have been uploaded to the Gene Expression Omnibus database (accession # GSE39082). The data were analyzed with the Tree View software and GSEA software, and expressed as a Heatmap (A) and Enrichment score (B).


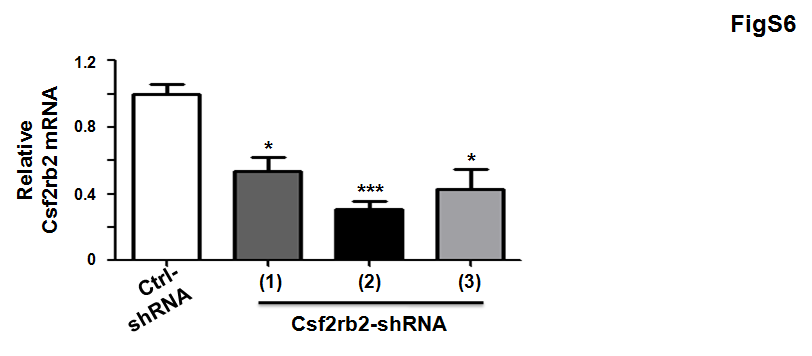


**Supplementary Figure S6. Knock down of Csf2rb2 expression by using Lentivirus-mediated shRNA transfection.** KSL cells purified from BM of C57BL/6 mice were transfected independently with 3 distinct shRNAs targeting Csf2rb2 and cultured under serum-free conditions for 6 days, followed qRT-PCR to determine Csf2rb2 expression (n = 3). *, P < 0.05, ***, P < 0.001.
